# Maternal Conditions and Cesarean Delivery in a High-Burden Southeast Louisiana Birth Cohort: Associations and Limitations of Administrative Delivery Data

**DOI:** 10.3390/healthcare14172779

**Published:** 2026-09-01

**Authors:** Eurydice Lang, Demetrice Smith, Marie Adorno, Gloria Giarratano, Mary Dioise Ramos

**Affiliations:** School of Nursing, Louisiana State University Health Sciences Center–New Orleans, New Orleans, LA 70112, USA; elang@lsuhsc.edu (E.L.); dsmi61@lsuhsc.edu (D.S.); madorn@lsuhsc.edu (M.A.); ggiarr@lsuhsc.edu (G.G.)

**Keywords:** maternal health, perinatal outcomes, hypertensive disorders of pregnancy, gestational diabetes, cesarean delivery, administrative data, health equity, quality improvement, care pathways, electronic health records

## Abstract

**Background**: Adverse perinatal outcomes impose a substantial burden on maternal health services, particularly in regions with a high prevalence of maternal medical conditions and marked health inequities. Population-specific evidence on how common maternal conditions relate to delivery outcomes remains limited for high-burden Southeast Louisiana. **Methods**: We conducted a retrospective cohort study of 7161 delivery encounters at an urban New Orleans hospital between 2020 and 2024, using electronic health record data from the LSU Health Clinical Data Warehouse. Multivariable logistic regression estimated adjusted associations of three non-overlapping exposure groups—gestational hypertension/preeclampsia (O13/O14), diabetes in pregnancy (predominantly gestational), and coded obesity—with cesarean delivery, ascertained from the recorded delivery-mode code and adjusted for age, race, ethnicity, and marital status; a composite of cesarean or preterm-labor coding was examined only as an exploratory secondary outcome. Because each encounter carried a single defined-condition code, the exposures were mutually exclusive, and their estimates describe separate exposure groups rather than the relative contributions of co-occurring conditions. **Results**: The cesarean rate ascertained from the delivery-mode code (11.9%) was well below the national benchmark (approximately 32%), indicating substantial administrative under-capture; such non-differential misclassification of the primary outcome would be expected to bias associations toward the null and limit the reliability of the effect estimates. Gestational hypertension/preeclampsia (adjusted odds ratio [aOR] 1.83, 95% confidence interval [CI] 1.53–2.19), diabetes (aOR 1.94, 95% CI 1.51–2.49), and coded obesity (aOR 1.41, 95% CI 1.05–1.88) were each associated with higher odds of cesarean delivery after adjustment for the available covariates. **Conclusions**: Because important clinical confounders (e.g., parity, prior cesarean delivery, multiple pregnancy, gestational age) were unavailable and the outcomes were not validated against clinical records, these associations should be interpreted as adjusted rather than independent. They are hypothesis-generating observations from administrative data rather than evidence for a risk-stratification tool, and they illustrate both the potential and the limits of routinely collected delivery data for monitoring maternal health services in high-burden settings.

## 1. Introduction

Adverse pregnancy outcomes remain a major public health concern in the United States [1], contributing to substantial maternal and neonatal morbidity and mortality and placing sustained demand on maternal health services [2,3]. Preterm birth affects approximately 10% of live births nationally [4] and is associated with neonatal intensive care admissions, long-term developmental sequelae, and considerable system-level cost [5]. Cesarean delivery is among the most common major surgical procedures performed in the United States, accounting for roughly one-third of all births [6]. Because these outcomes concentrate resource use—operative capacity, neonatal intensive care, and prolonged length of stay—a delivery system’s ability to anticipate and stratify risk has direct consequences for both patient safety and the efficient allocation of finite clinical resources.

Hypertensive disorders of pregnancy (HDP), diabetes mellitus, and obesity are among the most common maternal conditions and are each individually associated with adverse perinatal outcomes [7,8,9]. Population-specific evidence describing how these conditions relate to delivery outcomes within a single high-burden community, however, remains limited: most studies focus on a single maternal condition or a single outcome, and few describe several conditions together in the same underserved population [10]. Describing these associations is a useful first step for local quality-improvement and surveillance planning, although observational associations drawn from administrative data cannot by themselves determine which conditions should be prioritized in clinical care.

Southeast Louisiana presents a distinctive epidemiologic and health-system context. The region carries a disproportionate burden of maternal morbidity, driven by elevated rates of obesity, hypertension, and diabetes relative to national averages and compounded by persistent racial and socioeconomic inequities in access to and quality of maternal care [11]. Black women, who constitute the majority of the present cohort, experience preterm birth and maternal mortality at markedly higher rates nationally, even after accounting for socioeconomic factors [12,13]; in Louisiana the maternal mortality rate is among the highest in the nation, and hypertensive disorders have been identified as the leading cause of maternal death [11]. Despite this burden, population-specific evidence describing how maternal conditions relate to delivery outcomes in the region remains sparse.

Improving perinatal outcomes increasingly depends on system-level approaches to risk-appropriate care, structured risk stratification, standardized care pathways, and continuous quality improvement. Evidence-based obstetric safety bundles for hemorrhage and hypertension, such as those disseminated through the Alliance for Innovation on Maternal Health and state perinatal quality collaboratives, have produced measurable reductions in preventable maternal harm when implemented reliably [14,15]. Yet population-specific evidence describing how prevalent maternal conditions relate to delivery outcomes in such settings remains limited, particularly in the communities that carry the greatest burden.

This study addresses these gaps through a retrospective cohort analysis of 7161 delivery encounters at an urban hospital in New Orleans, Louisiana, between 2020 and 2024. Using multivariable logistic regression, we estimated the adjusted associations of three non-overlapping maternal-condition groups—gestational hypertension/preeclampsia, diabetes, and coded obesity—with cesarean delivery within a single high-burden, predominantly Black birth cohort, and examined a composite of cesarean or preterm-labor coding only as an exploratory secondary outcome. The analysis is intended to generate population-specific hypotheses about maternal risk and, more broadly, to illustrate how routinely collected delivery data can be used and where they fall short in monitoring maternal health services.

## 2. Materials and Methods

### 2.1. Study Design and Setting

This retrospective cohort study used electronic health record (EHR) data obtained from the LSU Health Clinical Data Warehouse, which aggregates records from the LCMC Health Epic system. The study site is a 261-bed urban hospital in New Orleans, Louisiana, that provides obstetric care to a diverse population reflective of the broader Southeast Louisiana region. The study period encompassed all deliveries from 1 January 2020 to 31 December 2024.

### 2.2. Ethical Considerations

The study protocol was reviewed and approved by the Institutional Review Board (IRB) prior to data access. Because the analysis used de-identified retrospective data, a waiver of informed consent was granted. Data were transmitted via a university-secured file-sharing platform and were accessible only to authorized study personnel; no direct patient contact occurred.

### 2.3. Participants and Eligibility

**Figure 1:**
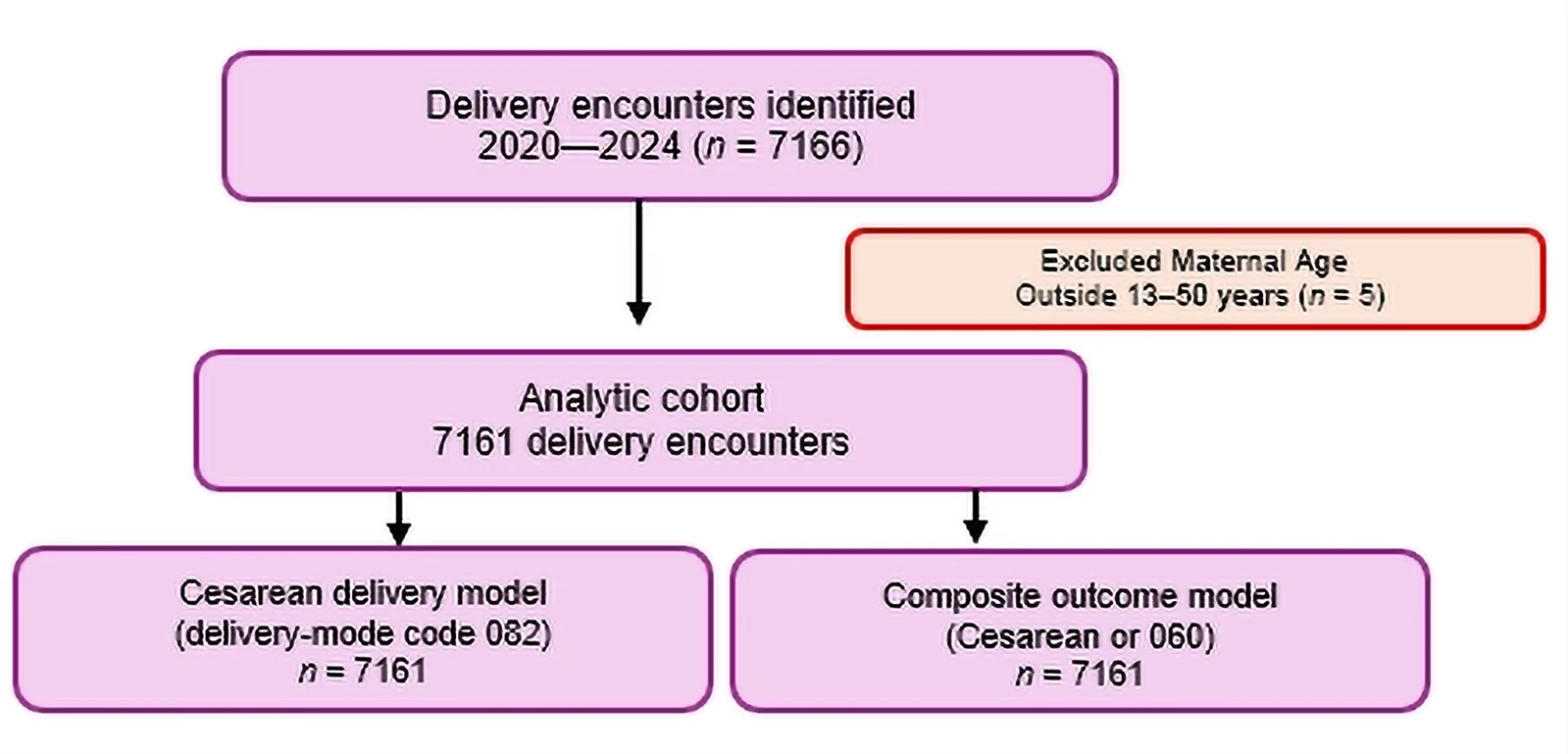
Participant flow through inclusion and the outcome-specific analyses. Exposures and outcomes were determined from ICD-10-CM diagnosis codes recorded during the delivery encounter; cesarean delivery was ascertained from the recorded delivery-mode code and the composite outcome from cesarean or preterm-labor coding; both models used the full analytic cohort (*n* = 7161).

Inclusion criteria were (1) delivery at the study hospital between 1 January 2020 and 31 December 2024, and (2) maternal age 13 to 50 years at delivery; no restrictions were placed on gestational age, parity, or comorbidity status. Exposures and outcomes were determined from ICD-10-CM diagnosis codes recorded during the delivery encounter, so each exposure and outcome was coded as present or absent for every included delivery; records with missing values on a model covariate were excluded from the corresponding analysis (complete-case analysis), and the analytic cohort comprised 7161 delivery encounters (all female, aged 13–50 years). The unit of analysis was the delivery encounter, and each encounter was treated as an independent observation. Deliveries were not linked across a woman’s pregnancies, so within-patient clustering for women who contributed more than one delivery during the period was not modeled; as discussed in the Limitations, the number of women with multiple deliveries within this five-year window is expected to be small and any resulting correlation modest, although mild underestimation of standard errors cannot be fully excluded. Participant flow and the analytic sample for each outcome are summarized in Figure 1.

### 2.4. Variables and Measures

**Maternal conditions.** Three maternal conditions were examined as predictors, each identified from International Classification of Diseases, Tenth Revision, Clinical Modification (ICD-10-CM) diagnosis codes recorded during the delivery encounter. HDP is an umbrella term for gestational hypertension (new-onset hypertension after 20 weeks without proteinuria or systemic features) and preeclampsia (hypertension with proteinuria or end-organ dysfunction); it was operationalized using O13 (gestational hypertension) and O14 (preeclampsia). Chronic hypertension (O10), superimposed preeclampsia (O11), and eclampsia (O15) were not included in this primary definition; because these categories carry elevated cesarean and preterm risk, their omission may have under-ascertained the full HDP spectrum, and we treat this as a classification limitation. Because the exposure captures only this subset, it is designated gestational hypertension/preeclampsia (O13/O14) throughout, and the term “HDP” is used only as shorthand for that coded subset rather than for the full clinical spectrum. Diabetes mellitus in pregnancy was identified using O24, which encompasses preexisting type 1 and type 2 diabetes and gestational diabetes mellitus (GDM); although pregestational and gestational diabetes differ in clinical profile and risk, the present analysis did not model the O24 subcategories separately (O24.0–O24.3 for pregestational and O24.4 for gestational diabetes). The two subtypes were tabulated separately (gestational, O24.4, n = 399; pregestational, other O24, n = 58) but were combined in the regression because pregestational cases were too few to model reliably; the diabetes estimate should therefore be read as reflecting predominantly gestational diabetes and combining the subtypes may obscure clinically meaningful heterogeneity between pregestational and gestational disease. Maternal obesity (body mass index [BMI] ≥ 30 kg/m^2^) was identified using O99.21 (obesity complicating pregnancy) and E66 (obesity, unspecified); because measured height and weight were not available in the de-identified dataset, obesity was ascertained from diagnosis codes alone, an approach known to under-record obesity substantially relative to measured BMI, which likely reduces sensitivity. In this analytic extract, each encounter carried a single defined-condition code, so HDP, diabetes, and obesity are mutually exclusive in these data (no encounter had more than one code); this precludes the co-occurrence, joint exposure, and interaction analyses and likely under-counts each condition.

**Delivery outcomes.** The primary outcome was cesarean delivery, ascertained from the recorded delivery mode code (O80, spontaneous vaginal delivery, vs. O82, cesarean delivery). Cesarean delivery is a mode of delivery that is frequently medically indicated rather than intrinsically adverse; because the dataset did not distinguish planned from unplanned or indicated from non-indicated cesarean, it is analyzed as a delivery outcome rather than as an inherently adverse event. We attempted to define preterm birth directly from gestational age using the recorded weeks-of-gestation (Z3A) codes; however, these codes were overwhelmingly antepartum (median 15 weeks; 98% below 37 weeks) and did not represent gestational age at delivery, so preterm birth could not be validly ascertained and was not modeled. The preterm-labor code (O60) was retained for descriptive purposes only. Because no ICD-10-PCS procedure codes were available, cesareans not captured by the delivery-mode code could not be recovered, and the resulting rate (11.9%) remained well below the approximately 32% national benchmark. A composite delivery outcome was defined as cesarean delivery or a preterm labor (O60) code. Because the O60 code captured only 2.6% of encounters and does not validly measure preterm birth, this composite adds little information beyond cesarean delivery and is numerically dominated by it; it is therefore treated as an exploratory secondary outcome, reported with caution and not as a primary result. No deliveries were coded for shoulder dystocia during the study period; because a complete absence of this intrapartum emergency in a cohort of this size is clinically implausible, the zero count was treated as an indicator of incomplete administrative capture, a data-quality observation rather than as a substantive null finding, and shoulder dystocia was not carried forward as an analyzable outcome.

**Covariates.** Demographic covariates comprised age at delivery (continuous), race (Black, White, Asian, or Other), Hispanic ethnicity (binary), and marital status (married versus unmarried). These were selected for their known associations with both the exposures and the outcomes and for their availability in the de-identified dataset.

### 2.5. Statistical Analysis

Descriptive statistics were summarized as mean (standard deviation) or median (interquartile range) for continuous variables and frequency (percentage) for categorical variables. For cesarean delivery (the primary outcome) and for the composite delivery outcome (examined only as an exploratory secondary analysis), a multivariable logistic regression model was fitted with the three maternal-condition groups entered simultaneously as predictors and the demographic covariates as adjustors; both crude (unadjusted) and adjusted odds ratios are reported. Because each encounter carried a single defined-condition code, the three exposures were mutually exclusive, so the estimates describe three separate exposure groups rather than the relative contributions of co-occurring conditions within individuals. Associations were reported as adjusted odds ratios (aORs) with 95% confidence intervals (CIs) and two-tailed *p*-values; statistical significance was set at α = 0.05. A complete-case approach was used. Because hypertensive disorders, diabetes, and obesity can co-occur clinically, the potential for multicollinearity among the three exposures was considered; these conditions represent distinct diagnostic constructs, and variance inflation factors (VIFs) were computed for the three exposures and were approximately 1.0 in every model, indicating no meaningful collinearity among HDP, diabetes, and obesity. Shoulder dystocia was not modeled: no cases were coded during the study period, and the zero count was interpreted as a data-quality signal rather than a true absence of events. Data management and cohort construction were performed in SAS version 9.4 [16], and all descriptive statistics and multivariable logistic regression models were conducted in SPSS version 29 [17].

## 3. Results

### 3.1. Sample Characteristics

The analytic cohort comprised 7161 delivery encounters occurring between 1 January 2020 and 31 December 2024 (Table 1). Mean age at delivery was 32 years (SD = 6.4; range 14–50). The cohort was predominantly Black (60.1%), followed by White (24.5%), Other racial groups (13.6%), and Asian (1.8%); most identified as non-Hispanic (85.1%). The majority were unmarried at delivery (67.9% single; 27.1% married).

### 3.2. Prevalence of Maternal Conditions and Adverse Outcomes

Among the 7161 delivery encounters, HDP was recorded in 1229 (17.2%), diabetes in 457 (6.4%; gestational 399 [5.6%], pregestational 58 [0.8%]), and obesity in 462 (6.5%); because the extract recorded one defined-condition code per encounter, no encounter had more than one of these conditions. Cesarean delivery by the delivery-mode code occurred in 855 encounters (11.9%) and a preterm-labor (O60) code in 187 (2.6%). No cases of shoulder dystocia (O66.0) were identified during the study period. The composite delivery outcome (cesarean or O60) occurred in 1035 encounters (14.5%). The cesarean rate (11.9%) remained well below the contemporary national benchmark (approximately 32%), consistent with administrative under-capture that the available data could not correct. Recorded gestational-age (Z3A) codes were antepartum (median 15 weeks; 98% below 37 weeks) and could not define preterm birth at delivery, which was therefore not modeled; this under-ascertainment should be borne in mind when interpreting the estimates that follow.

### 3.3. Multivariable Associations

Multivariable results are presented in Table 2. Preterm birth was not modeled because the recorded gestational-age codes were antepartum and could not define preterm birth at delivery.

For cesarean delivery, all three maternal conditions were associated with higher adjusted odds. The aOR for HDP was 1.83 (95% CI 1.53–2.19, *p* < 0.001; crude OR 1.66); the aOR for diabetes was 1.94 (95% CI 1.51–2.49, *p* < 0.001; crude OR 1.96); the aOR for obesity was 1.41 (95% CI 1.05–1.88, *p* = 0.02).

In the exploratory secondary analysis of the composite outcome, gestational hypertension/preeclampsia (aOR 1.26, 95% CI 1.06–1.50, *p* = 0.009) and diabetes (aOR 1.44, 95% CI 1.13–1.84, *p* = 0.004) were associated with the composite after adjustment, whereas coded obesity was not (aOR 0.95, 95% CI 0.71–1.26, *p* = 0.71); because the composite is dominated by cesarean delivery and incorporates a preterm-labor code that does not validly measure preterm birth, these estimates are reported only as secondary and hypothesis-generating. Variance inflation factors for the three exposures were approximately 1.0 in both models, confirming no collinearity among HDP, diabetes, and obesity; indeed, because the analytic extract recorded a single defined condition per encounter, the three exposures were mutually exclusive in these data.

## 4. Discussion

In this retrospective cohort of 7161 deliveries from a high-burden, predominantly Black population in Southeast Louisiana, gestational hypertension/preeclampsia, diabetes mellitus, and coded obesity were each associated, after adjustment for available demographic covariates, with higher odds of cesarean delivery; in an exploratory secondary analysis, HDP and diabetes were also associated with the composite outcome. Because the exposures were mutually exclusive in this administrative extract, these estimates describe three separate exposure groups rather than the relative contributions of co-occurring conditions, and because the primary outcome was substantially under-captured and was not validated against clinical records, the findings are best read as hypothesis-generating associations rather than as evidence supporting a risk-stratification tool. Interpreted cautiously through a health-services lens, they point to HDP and diabetes as conditions warranting attention in future risk-stratification and quality-improvement research, and they illustrate both the utility and the limitations of routinely collected delivery data for this purpose.

### 4.1. Hypertensive Disorders and Diabetes as Candidate Priorities for Future Risk-Stratification Research

The association of HDP with cesarean delivery (aOR 1.83) is consistent with established pathophysiology: preeclampsia frequently necessitates medically indicated delivery before spontaneous labor, particularly with worsening end-organ dysfunction or severe-range blood pressure. Population-based studies have similarly identified preeclampsia as a leading driver of iatrogenic preterm and unplanned operative delivery, with cesarean rates among affected women ranging from 36% to 63% depending on gestational age at onset and severity [18]. The association of diabetes (aOR 1.94) is likewise well supported: diabetes elevates the risk of macrosomia predisposing to cephalopelvic disproportion, failed induction, and operative delivery and diabetes-associated placental dysfunction may prompt earlier intervention via induction or cesarean [19]. HDP and diabetes showed adjusted associations of similar magnitude with the composite outcome (aOR 1.26 and 1.44, respectively); because these rounded estimates were not formally compared, this similarity does not establish equivalence, though both conditions merit attention in surveillance and escalation planning.

From a delivery-system standpoint, these associations are consistent with, but do not by themselves establish, a role for HDP and diabetes as anchor conditions in structured perinatal risk stratification; because this study did not evaluate discrimination, calibration, internal validation, or clinical utility, it cannot demonstrate that flagging these conditions would improve outcomes. They do, however, motivate hypotheses worth testing in appropriately designed work—for example, whether EHR-based flags that identify affected patients at triage, predefined escalation thresholds (such as severe-range blood pressure ≥ 160/110 mmHg), standing order sets, and clear criteria for specialist consultation and risk-appropriate level-of-care assignment improve care. Prior evidence indicates that reliable implementation of hypertension and related safety bundles, rather than ad hoc response, is the mechanism through which such high-risk conditions translate into improved outcomes [14,15].

### 4.2. Reinterpreting the Role of Obesity for Service Design

Obesity was associated with higher odds of cesarean delivery after adjustment (aOR 1.41) but showed no association with the composite outcome. These estimates must be read against a structural feature of the analytic extract: because each encounter carried a single defined-condition code, obesity could not co-occur with HDP or diabetes in these data, so the model cannot separate an obesity-specific effect from the effect of unrecorded comorbidities, and no mediation interpretation is possible [20,21]. For service design, the greatest reduction in obesity-attributable perinatal risk is likely to come from intensive detection and management of HDP and GDM in patients with obesity, rather than from weight-focused intervention during an index pregnancy. This interpretation should be drawn cautiously, however, given that obesity was ascertained from diagnosis codes rather than measured BMI values.

### 4.3. Equity-Centered Implications for Hospitals Serving High-Risk Populations

The cohort’s racial composition, 60.1% Black, mirrors the population most affected by maternal health inequities in the United States and in Louisiana specifically, where Black women experience markedly higher maternal mortality and where HDP has been identified as the leading cause of maternal death [11]. The present analysis did not compare outcomes across racial or ethnic groups and therefore does not by itself demonstrate a within-cohort disparity; the equity implications discussed here rest on established external evidence rather than on stratified analyses of these data, which remain a priority for future work. The finding that HDP and diabetes show the largest adjusted associations with cesarean delivery among the conditions examined in this predominantly Black cohort reinforces the case for equity-focused, system-level intervention. Closing outcome gaps in such settings depends less on individual risk modification than on the structural reliability of care: equitable access to antihypertensive therapy and timely escalation, standardized postpartum blood-pressure surveillance, culturally concordant patient education and communication, and outcome monitoring stratified by race and ethnicity so that disparities in process and outcome become visible to quality-improvement teams. Hospitals that serve a disproportionate share of high-risk patients are precisely the settings in which reliable, protocol-driven maternal care has the greatest potential to narrow inequities. Culturally concordant care in these settings extends beyond the individual patient to the family and community context of pregnancy and the postpartum period; community-engaged, family-inclusive approaches—including the involvement of partners and fathers in perinatal education and support—are increasingly recognized as levers for improving maternal and infant health in African American communities [22].

### 4.4. Implications for Maternal Health Services, Care Pathways, and Quality Improvement

The findings suggest several directions for maternal health services in high-burden settings that warrant evaluation in future work rather than immediate implementation on the basis of these data. First, the observation that HDP and diabetes carry the largest adjusted associations motivates testing whether system-level risk stratification—EHR-based flags and triage criteria that identify these conditions early in the care episode so that surveillance intensity is matched to risk rather than applied uniformly—improves outcomes. Second, standardized care pathways and safety bundles for hypertension and diabetes should be implemented reliably and audited for fidelity; for hypertension this includes structured blood-pressure monitoring, prompt treatment of severe-range values, and explicit escalation protocols [15], and for diabetes it includes intrapartum glucose management, insulin protocols, and neonatal hypoglycemia surveillance, together with discharge education addressing the substantial lifetime risk of type 2 diabetes following GDM [8]. Third, because reliable execution of these pathways depends on the frontline workforce— particularly nurses, who perform much of the surveillance and escalation at the bedside—adequate staffing, training, and team-based protocols are themselves system-level determinants of outcome. Fourth, given the concentration of adverse outcomes among Black women in this and comparable populations, services should embed equity into quality improvement through stratified outcome monitoring, anti-bias training, and attention to structural barriers to access and postpartum follow-up. Finally, the null finding for coded obesity should not by itself guide resource allocation, given the under-capture of obesity in these data; it does, however, raise the hypothesis—worth testing with measured BMI and co-occurring diagnoses—that detection and management of HDP and GDM in patients with obesity may matter more than weight-focused goals during an index pregnancy.

### 4.5. Why Preterm Birth Could Not Be Validly Ascertained

Preterm birth could not be modeled in this cohort. We first attempted to define it directly from gestational age using the recorded weeks-of-gestation (Z3A) codes, as a valid ascertainment requires; however, these codes were overwhelmingly antepartum (median 15 weeks, with 98% below 37 weeks) and therefore did not represent gestational age at delivery. The only alternative, the single preterm-labor code (O60), captured just 2.6% of encounters, far below the approximately 10% national benchmark [4], and reflects the same single-code under-ascertainment that affects cesarean delivery. Rather than report associations for an outcome the data could not validly measure, we omitted preterm birth from the regression analyses. This limitation is itself informative: it shows that routinely coded gestational-age data, though present, may not correspond to the delivery event and cannot be assumed valid for perinatal outcome ascertainment without verification.

### 4.6. Strengths and Limitations

This study has several strengths. It draws on a large, real-world birth cohort from a population that is both high-burden and underrepresented in the perinatal literature, and it examines three common maternal conditions within a single multivariable framework. Because the exposures were mutually exclusive in this extract, the analysis compares three separate exposure groups rather than the relative contributions of co-occurring conditions within individuals. Its use of routinely collected delivery data also demonstrates how existing health-system information can be repurposed for population-specific description and hypothesis generation.

Several limitations should nonetheless be weighed in interpreting these findings. First, as a single-site analysis conducted at one urban hospital, the results reflect the case mix, referral patterns, payer profile, and clinical practices of that facility and may not generalize to other settings, particularly the rural communities of Southeast Louisiana, where access to risk-appropriate maternal care differs markedly. Multi-site studies spanning urban and rural facilities are needed to establish external validity.

Second, exposures and outcomes were ascertained from ICD-10-CM codes, which are generated for billing and administrative purposes rather than research. Administrative coding is subject to incomplete and inconsistent documentation and cannot capture clinical severity or control; for example, it does not distinguish gestational hypertension from severe preeclampsia, well-controlled from poorly controlled diabetes, or measured BMI strata. The implausibly low observed cesarean rate (11.9%) relative to the national benchmark, together with gestational-age codes too antepartum to ascertain preterm birth, strongly suggest under-determination of these outcomes; if such misclassification is non-differential with respect to exposure, the resulting bias would generally attenuate associations toward the null and may partly explain the null findings for preterm delivery and for obesity. Validation of cesarean and preterm-birth ascertainment against delivery records, birth certificates, or another clinical reference source was recommended during peer review but could not be performed within the de-identified extract; it remains an essential next step, and until it is completed these associations should not be used to guide service allocation or clinical prioritization. This under-capture affects the primary outcome specifically—the observed cesarean rate is roughly a third of the expected value, so the outcome is likely subject to substantial, largely non-differential misclassification that would bias the effect estimates toward the null and limit their reliability.

Third, no cases of shoulder dystocia (O66.0) were identified. Shoulder dystocia is an uncommon intrapartum emergency (estimated incidence 0.2% to 3% of vaginal deliveries), and with a low coded vaginal-delivery volume in this cohort, true events may have gone uncoded or been captured under alternative codes. The absence of these data precludes any conclusion about this outcome and again stresses the limits of single-code administrative determination for acute intrapartum events; supplementing administrative data with delivery records and nursing documentation would improve capture.

Fourth, the available covariate set was limited to a small number of demographic variables. Clinically important confounders were unavailable in the de-identified dataset, including parity and prior cesarean delivery (a major determinant of mode of delivery), multiple (twin or higher-order) pregnancy, fetal presentation, induction of labor, gestational age at delivery, estimated fetal size (e.g., macrosomia), measured BMI, gestational age at entry to prenatal care and adequacy of prenatal care, insurance or payer status, provider type (e.g., midwife versus obstetrician), and specifics of intrapartum management. Accordingly, the associations reported here should be regarded as adjusted for the available demographic covariates but not as independent of clinical confounders. A related limitation concerns the unit of analysis: because deliveries were analyzed at the encounter level without linkage across a woman’s pregnancies, encounters contributed by women who delivered more than once during the study period were treated as independent observations, which could modestly narrow the reported confidence intervals, although the number of such repeat deliveries within the five-year window is likely small. Residual confounding by these factors is therefore likely, and the reported associations should be interpreted as adjusted but not fully confounding-controlled. The likely direction of this residual confounding differs by factor. Nulliparity is associated with both a higher risk of preeclampsia and a greater likelihood of cesarean delivery, so if HDP-affected women in this cohort were disproportionately nulliparous, the inability to adjust for parity could have inflated the HDP–cesarean association; similarly, a higher prevalence of prior cesarean delivery—a strong indication for repeat cesarean—among women with diabetes or HDP could bias both estimates upward. Working in the opposite direction, the absence of measures of disease severity and glycemic control (which group well-controlled and poorly controlled cases together) and of prenatal-care adequacy would tend to attenuate the true associations toward the null. On balance, the HDP and diabetes estimates reported here are best regarded as potentially subject to modest upward bias from unmeasured obstetric history, partially offset by non-differential misclassification of severity; the net direction cannot be determined from the available data.

The composite outcome aggregates events of differing clinical significance and was dominated by cesarean delivery, so associations with the composite largely reflect that outcome. The complete-case approach may introduce bias if data were not missing completely at random, although the large sample and relatively complete demographic data mitigate this concern. The study period (2020–2024) overlaps the COVID-19 pandemic, which affected delivery volumes, staffing, and patterns of care and may have influenced both outcomes and their documentation. During 2020 and 2021 in particular, altered prenatal-care delivery (including expanded telehealth), fluctuations in delivery volume and staffing, and shifting documentation and coding practices could have affected both the occurrence and the administrative capture of the study outcomes. Because these disruptions were concentrated early in the study window, they may have contributed to the observed under-ascertainment; a year-stratified sensitivity analysis contrasting the pandemic-affected years (2020–2021) with 2022–2024 would help quantify any temporal effect and is a priority for future work with this cohort. Finally, the observational design precludes causal inference, and the data capture diagnoses and outcomes but not the processes of care delivered, limiting direct conclusions about which service-level interventions would most improve outcomes.

## 5. Conclusions

In a large, predominantly Black birth cohort from a high-burden Southeast Louisiana hospital, hypertensive disorders of pregnancy, diabetes mellitus, and obesity were each associated with higher odds of cesarean delivery after adjustment for available covariates, with HDP and diabetes additionally associated with the composite outcome in an exploratory secondary analysis; given the unmeasured clinical confounders discussed above, these associations should be read as adjusted rather than independent of clinical confounders. These findings identify HDP, diabetes, and coded obesity as conditions associated with cesarean delivery and as candidate topics for future research on standardized care pathways and equity-focused quality improvement in hospitals serving high-risk populations; because outcome ascertainment was limited by the administrative data (cesarean under-capture and the inability to validly measure preterm birth) and because the extract could not capture co-occurring conditions, these associations are best read as hypothesis-generating and require confirmation in data with valid outcome and comorbidity ascertainment. They also demonstrate both the promise and the limits of routinely collected delivery data for monitoring maternal health services: the same administrative records that enable population-specific analysis under-capture key outcomes, a constraint that must be addressed for such data to support reliable surveillance. Future work should pursue multi-site, urban–rural designs with validated outcome confirmation, examine the mediating pathways linking obesity to adverse outcomes, and evaluate whether reliable implementation of risk-stratified care pathways and safety bundles narrows the maternal outcome inequities that persist in this region and others like it.

## Figures and Tables

**Table 1:** Summary of demographic characteristics.

Characteristic	Complete Cohort (*n* = 7161)
**Age at delivery, years**	
Mean (SD)	32.0 (6.4)
Median (IQR)	32.0 (27.0–37.0)
Range	14–50
**Race, *n* (%)**	
Black	4305 (60.1)
White	1757 (24.5)
Asian	128 (1.8)
Other	971 (13.6)
**Ethnicity, *n* (%)**	
Hispanic	1068 (14.9)
Non-Hispanic	6093 (85.1)
**Marital status, *n* (%)**	
Single	4859 (67.9)
Married	1944 (27.1)
Other	358 (5.0)
**Maternal conditions, *n* (%)**	
HDP (O13/O14)	1229 (17.2)
Diabetes (O24)	457 (6.4)
Gestational (O24.4)	399 (5.6)
Pregestational (other O24)	58 (0.8)
Obesity (O99.21x/E66)	462 (6.5)

IQR = interquartile range; SD = standard deviation. The “Other” marital-status category combines life partner, divorced, legally separated, widowed, and other responses.

**Table 2:** Crude and adjusted associations between maternal conditions and delivery outcomes (n = 7161).

Outcome	Maternal Condition	Crude OR (95% CI)	Adjusted OR (95% CI)	*p*-Value
**Cesarean delivery**				
	HDP	1.66 (1.40–1.97)	1.83 (1.53–2.19)	<0.001
	Diabetes	1.96 (1.54–2.50)	1.94 (1.51–2.49)	<0.001
	Obesity	1.11 (0.84–1.47)	1.41 (1.05–1.88)	0.02
**Composite delivery outcome**				
	HDP	1.26 (1.06–1.48)	1.26 (1.06–1.50)	0.009
	Diabetes	1.54 (1.21–1.96)	1.44 (1.13–1.84)	0.004
	Obesity	0.88 (0.66–1.16)	0.95 (0.71–1.26)	0.71

Models adjusted for age at delivery, grouped race, Hispanic ethnicity, and marital status; coefficients for these covariates were estimated. The composite delivery outcome is an exploratory secondary analysis. OR = odds ratio; CI = confidence interval. Variance inflation factors for the three exposures were approximately 1.0. Preterm birth was not modeled because the recorded gestational-age codes were antepartum.

## Data Availability

The data presented in this study are not publicly available because they are derived from de-identified electronic health records and are subject to institutional data-use restrictions. De-identified data may be available from the corresponding author upon reasonable request and with the permission of LSUHSC NO.

## Abbreviations

The following abbreviations are used in this manuscript: aOR, adjusted odds ratio; BMI, body mass index; CI, confidence interval; EHR, electronic health record; GDM, gestational diabetes mellitus; HDP, hypertensive disorders of pregnancy; ICD-10-CM, International Classification of Diseases, Tenth Revision, Clinical Modification.
